# Socioeconomic status is associated with global diabetes prevalence

**DOI:** 10.18632/oncotarget.17902

**Published:** 2017-05-16

**Authors:** Zhiye Xu, Dan Yu, Xueyao Yin, Fenping Zheng, Hong Li

**Affiliations:** ^1^ Department of Endocrinology, The Affiliated Sir Run Run Shaw Hospital, School of Medicine, Zhejiang University, 310016, Hangzhou, Zhejiang Province, China; ^2^ Department of Endocrinology, Zhejiang Hospital, 310013, Hangzhou, Zhejiang Province, China

**Keywords:** diabetes, prevalence, socioeconomic status, human development index (HDI)

## Abstract

The incidence of diabetes is increasing globally. We investigated the relationship between diabetes prevalence and patient socioeconomic status across multiple countries. We searched PubMed to identify population-based surveys reporting diabetes prevalence between 1990 and May 2016. Search results were filtered, and Human Development Index (HDI) values from the United Nations Development Programme were used to assess socioeconomic status for a given nation. Our analysis included 45 national surveys from 32 countries. Diabetes prevalence was positively correlated with national HDI (*r* = 0.421 *P* = 0.041) in developing countries, and negatively correlated with HDI (*r* = −0.442 *P* = 0.045) in developed countries. Diabetes prevalence trends were the same in women and men, although men were associated with increased diabetes risk in developed countries (*r* = 0.459 *P* = 0.048). Thus, diabetes prevalence rises with increasing HDI in developing countries, and this is reversed in developed countries. Ours is the first study to investigate the relationship between diabetes and socioeconomic status at global level using HDI values. These results will aid in evaluating global diabetes prevalence and risk with respect to patient socioeconomic status, and will be useful in the development of policies that help reduce disease incidence.

## INTRODUCTION

Diabetes case numbers rose worldwide from 285 million adults in 2009 to 382 million in 2013, with a projected 471 million by 2030 [1, 2]. Additional diabetes risk factors outside those already confirmed, such as overweight, obesity, and high-calorie diet, must be better characterized. Some groups reported that low socioeconomic status may indicate higher diabetes risk [3, 4], and lower-income patients are less likely to achieve therapeutic goals [5]. However, conflicting studies from different countries reported either no association between socioeconomic status and diabetes [6], or that higher socioeconomic status is related to increased type 2 diabetes risk [7]. These inconsistencies may result in part from changing socioeconomic statuses within these countries, and must be further explored.

To our knowledge, while researchers have investigated relationships between diabetes prevalence and socioeconomic status in specific countries or regions, this interaction has yet to be studied at a global level. We investigated possible global correlations between diabetes prevalence and socioeconomic status according to the Human Development Index (HDI).

## RESULTS

### Study selection

**Figure 1:**
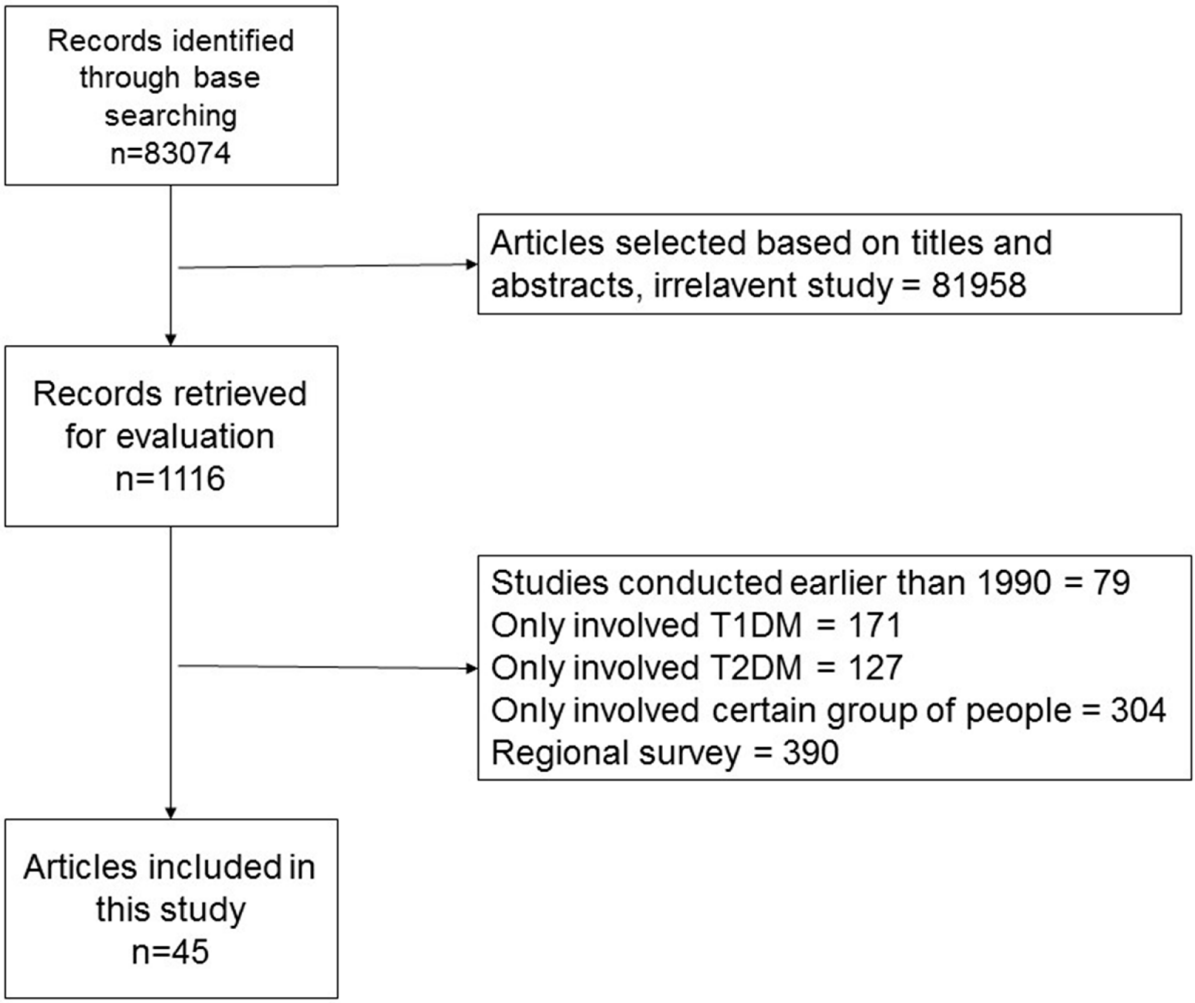
Search flowchart

Our initial search identified 83,074 records (Figure 1). Following screening, we included 44 articles in our analysis, representing 45 studies from 32 different countries (Supplementary Table 1). Approximately half of the studies (48.9%; 22/45) were conducted in Asia, and 26.7% (12/45) in Europe. No studies from South America met our inclusion criteria. According to HDI, 24/45 studies were conducted in developing countries, and 21/45 in developed countries. Investigator agreement with respect to study inclusion was excellent (Kappa statistic, 0.883).

### Diabetes prevalence and national HDI

**Figure 2:**
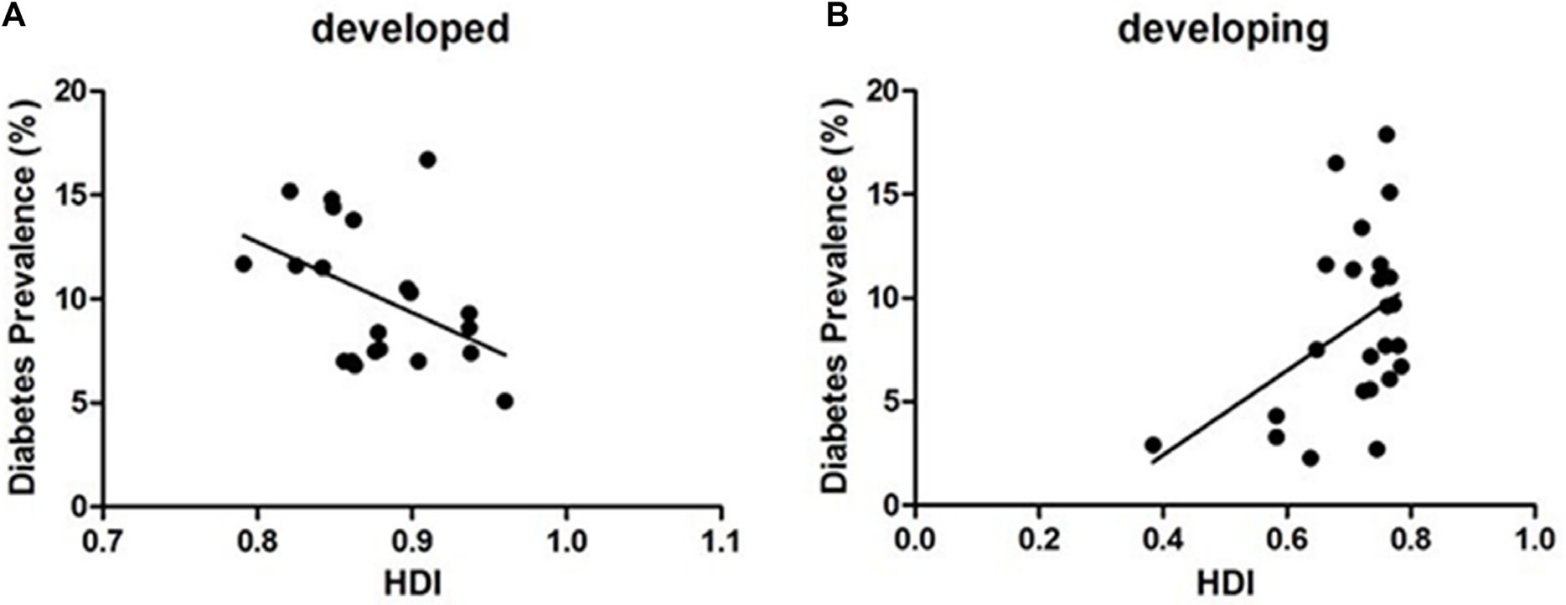
The correlation between diabetes prevalence and HDI in developed countries (**A**) (*r* = −0.442 *P* = 0.045) and developing countries (**B**) (*r* = 0.421, *P* = 0.041).

Global diabetes prevalence was 9.34% according to our study. In developing countries, prevalence was 8.67% and increased with increasing national HDI (*r* = 0.421, *P* = 0.041). Diabetes prevalence was 10.10% in developed countries and decreased with increasing national HDI (*r* = −0.442 *P* = 0.045) (Figure 2). There was no prevalence difference between developing and developed countries according to Student's t test (*P* = 0.22).

### Diabetes prevalence within study years

**Figure 3:**
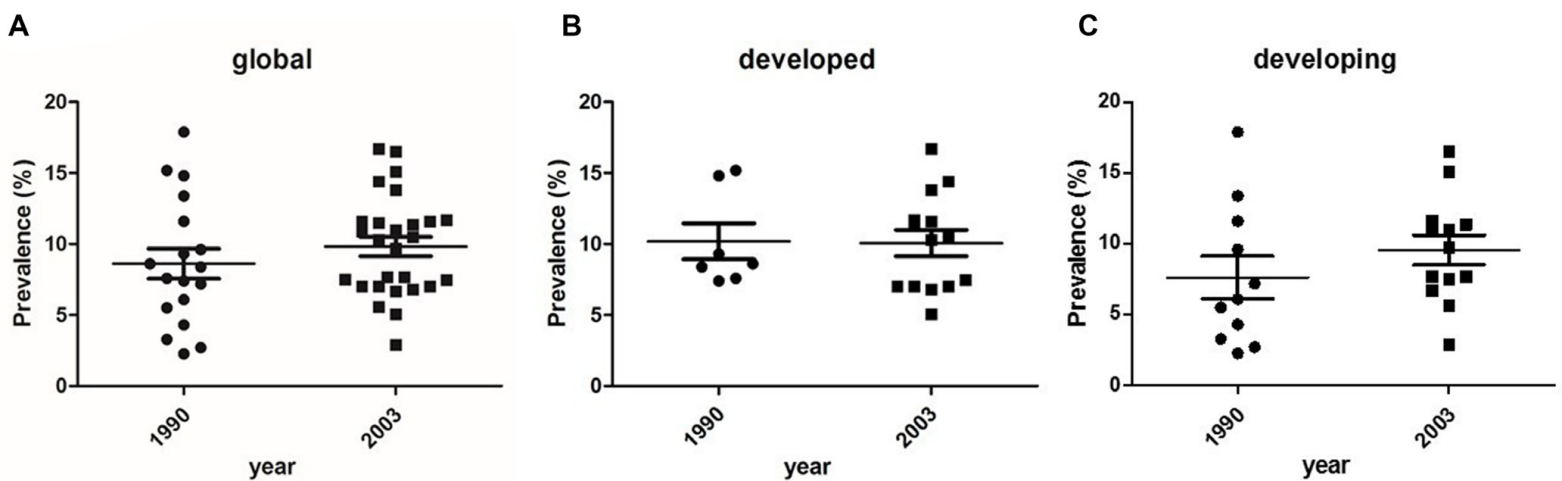
The scatter plots of diabetes prevalence during the two time periods (1990–2003 and 2003–present) (**A**) world: *P* = 0.32, t = 1.00; (**B**) developed countries: *P* = 0.94, t = 0.08; (**C**) developing countries: *P* = 0.29, t = 1.083.

Scatter plot results suggested that there was no difference between diabetes prevalence during the two time periods (1990–2003 and 2003–present) (world: *P* = 0.32, t = 1.00; developing countries: *P* = 0.29, t = 1.083; developed countries: *P* = 0.94, t = 0.08) (Figure 3).

### Diabetes prevalence by gender

**Figure 4:**
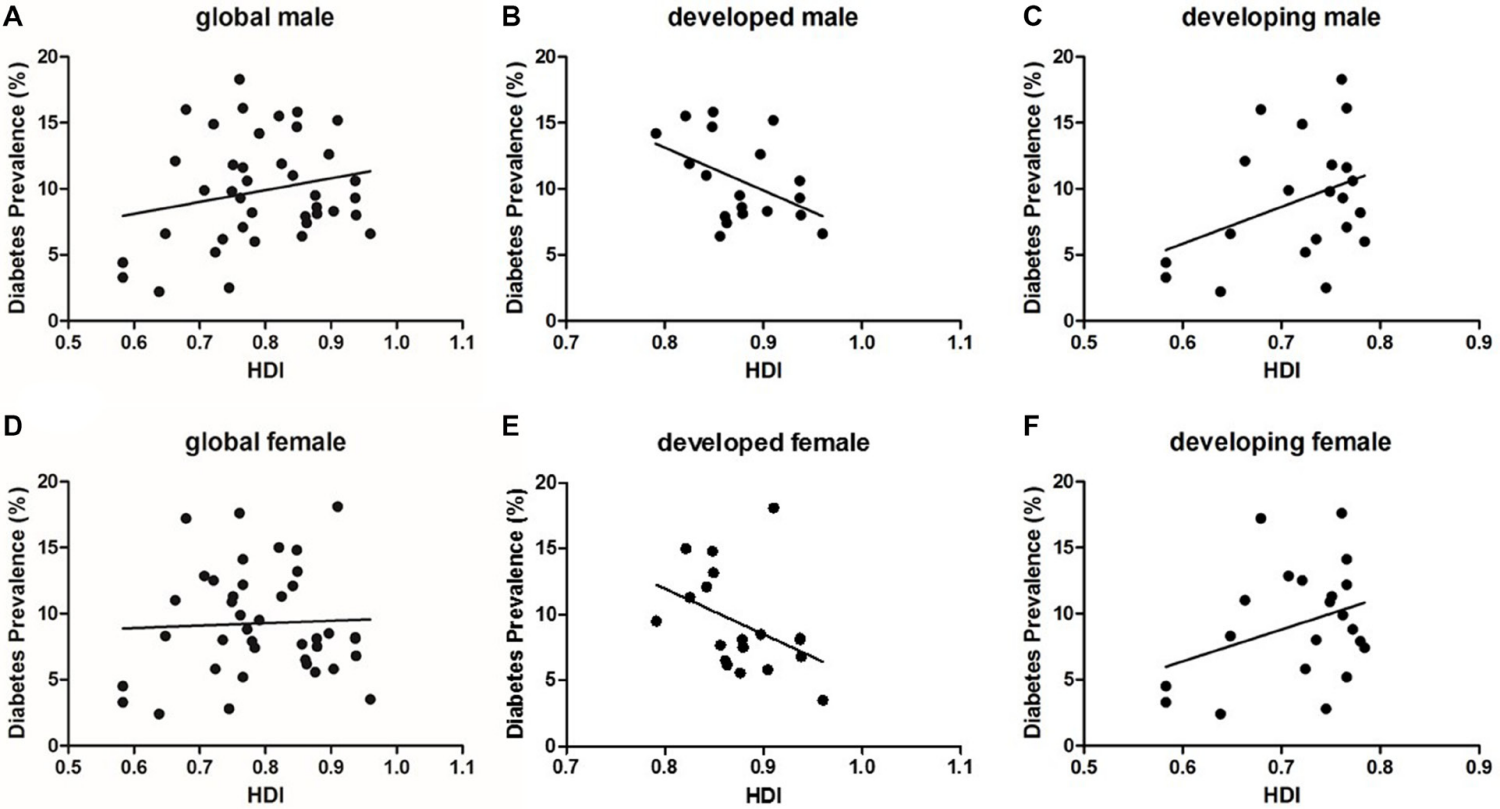
The correlation between diabetes prevalence and HDI by gender (**A**) global male (*P* = 0.18); (**B**) developed male (*P* = 0.048); (**C**) developing male (*P* = 0.095); (**D**) global female (*P* = 0.78); (**E**) developed female (*P* = 0.080); (**F**) developing female (*P* = 0.128).

Global diabetes prevalence was 9.84% in men and 9.26% in women (no significant difference). Scatter plots indicated no correlation between HDI and diabetes prevalence in men (*P* = 0.18) or women (*P* = 0.79). The same trends were observed in developing countries. However, intra-group comparisons revealed a correlation between HDI and prevalence in men in developed countries (r = 0.459 *P* = 0.048) (Figure 4).

### Study methods

**Figure 5:**
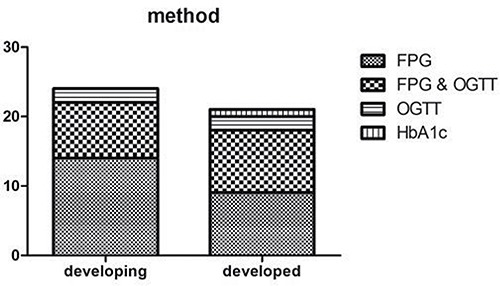
Study methods difference between developing and developed countries (*P* = 0.582)

All included studies reported definite diagnostic methods. The main methods to diagnose diabetes are fasting plasma glucose (FPG) and oral glucose tolerance test (OGTT). FPG was applied in 40/45 included studies, and OGTT was performed in 21/45 studies; 17 studies used both methods. Three studies assayed glycated hemoglobin (HbA1c) levels to diagnose diabetes. Diagnostic methods did not differ between developing and developed countries *P* = 0.582 (Figure 5).

## DISCUSSION

Regional studies have assessed the effects of socioeconomic factors on diabetes prevalence. A 2003 study in Turin, Italy found that age-adjusted diabetes prevalence was higher among those with lower income and/or education levels [8]. A seven-year-long study in Tianjin, China suggested that lower income and retirement were associated with increased impaired fasting glucose (IFG) and type 2 diabetes risk [9]. Another survey study showed that socioeconomic status was a diabetes risk factor for women, and possibly for men [10]. While regional studies are of great value to clinicians, relationships between socioeconomic status and diabetes prevalence must also be assessed at a global level.

HDI is a commonly used national socioeconomic status indicator, and may be the gold standard for international comparisons. Our analysis of 45 studies from 32 different countries found that diabetes prevalence in developing countries increased with increasing HDI. This was consistent with previous studies [11]. However, this trend was reversed in developed countries, where diabetes prevalence decreased with increasing HDI. These findings support the notion that socioeconomic status is directly related to diabetes prevalence worldwide. Economic changes in developing countries over the last three decades have dramatically improved living standards, and obesity and sedentary lifestyles are becoming more common [12]. Adult diabetes cases are expected to increase by 69% between 2010 and 2030 in developing countries, and by 20% in developed countries, whereas total adult populations are expected to rise by only 36% and 2%, respectively [1].

Patients in developed countries are more likely than those in developing countries to have access to high quality medical services and health education systems. Most countries that spend ≥ $2000 USD per capita on diabetes are developed, while nearly all those spending ≤ $1000 USD per capita are developing [13]. The United States of America alone accounts for more than half of global diabetes-related expenditures, while < 10% of global diabetes costs will be spent in low- and middle-income countries where approximately 70% of diabetes patients lived in 2010 [13]. Additionally, individuals who received fewer years of education were at higher risk for diabetes [14]. Our results are consistent with these findings, although we found no significant difference between diabetes prevalence in developing versus developed countries. This suggests that diabetes incidences are increasing worldwide, in both developing and developed countries.

While diabetes trends were generally the same for women and men in developing and developed countries, men were associated with increased diabetes risk in developed countries with statistic difference. Previous studies suggested gender may impact socioeconomic status, lifestyle, and health-related behavior, and thus also diabetes risk [11]. For example, that prevalence of diabetes is directly related to deprivation, especially for women in Italy [15]. A 2011 meta-analysis also found that socioeconomic status was lower in women with type 2 diabetes than in men [16].

Our results suggested that there was no up- or downtrend in diabetes prevalence globally or in developed or developing countries across the study years. This result is inconsistent with widely accepted findings that diabetes prevalence has increased globally over the past several years [1, 2]. This discrepancy might be attributed to publication bias, in that countries with high diabetes incidences may be more likely to publish diabetes-related survey and research results.

Previous studies indicated that diabetes prevalence within a given population might vary to some extent, depending on diagnostic criteria [17]. In our study, four kinds of diagnostic criteria were used to diagnose diabetes, with no differences observed between developed and developing countries. Despite medical infrastructure disadvantages in developing countries, clinicians in these countries applied up-to-date guidelines in diabetes treatment practices.

Ours is the first study concerning the global relationship between diabetes prevalence and socio-economic status. All studies included in our analysis were national, rather than regional, investigations. We chose HDI as a measurement of socioeconomic status, as HDI is widely used to make development-related comparisons between countries. Compared with other indices such as gross domestic product (GDP) which only related to economy, HDI is more comprehensive and comparable with three aspects that containing economy, health and education. We used an exhaustive search strategy to identify all relevant literature. Two investigators independently performed study eligibility assessments and data extraction, with discrepancies resolved by a third reviewer.

A main limitation of our study was that data was unavailable for many countries especially those located on the African and South American continents. This limited the accuracy and reliability of our findings. In addition, our study was restricted to publications written in English, which undoubtedly excluded studies from certain countries.

In conclusion, we found that diabetes prevalence increased with increasing HDI in developing countries, and decreased with increasing HDI in developed countries. Further research is needed to characterize relationships between diabetes prevalence and socioeconomic status in individual countries. Our results will aid in evaluating global diabetes prevalence and risk with respect to patient socioeconomic status, and will be useful in the development of policies that help reduce disease incidence.

## MATERIALS AND METHODS

### Study selection

We performed a literature search in PubMed (1948 to February 2016) with following terms: (diabetes) AND (epidemic OR prevalence OR population OR morbidity OR incidence). Studies identified in the initial search were screened and evaluated using the following inclusion criteria: (i) national population-based survey; (ii) conducted in an adult population; (iii) definite diagnostic method, criteria, and data; (iv) nation has HDI data; (v) published in English; (vi) no self-reported surveys. For publications retrieved for detailed examination (1116 articles), two researchers (Xueyao Yin & Dan Yu) independently filled in standardized forms with predefined inclusion criteria. In cases of any study that caused disagreement for inclusion, the corresponding author independently filled in the form and consensus was reached. To reduce overlapping, we included original studies used in multiple publications only once, giving preference to studies with larger sample sizes and more recent publication dates.

### Data extraction

Two researchers (Zhiye Xu and Dan Yu) independently extracted the following data from the studies using Microsoft Excel (2007 edition; Microsoft, Redmond, WA): title, author, country, publication year, years of data collection, sample size, patient ages, HDI, diabetes prevalence, diagnostic method, diagnostic criteria. If both crude and adjusted prevalence were stated in the article, the latter was included.

### HDI estimation

In this study, HDI was chosen as a socioeconomic development indicator. HDI is a summary measure of human development, which is a composite index of three basic dimensions, including income index (based on GDP per capita adjusted for purchasing-power parity, US$), health index (based on life expectancy at birth), and education index (based on a combination of adult literacy rate and primary to tertiary education enrollment rates). These indices are calculated by the World Bank and International Monetary Fund, the United Nations (UN) Department of Economic and Social Affairs, and the United Nations Educational, Scientific, and Cultural Organization. According to the UN, countries with high HDI scores (≥0.788) are regarded as developed, while all others are defined as developing [18].

Data for UN member states from 1990–2013 were obtained from the UN Development Programme database [19]. Due to a lack of pre-1990 HDI data, surveys conducted before 1990 were excluded. If a study was performed over > 1 year, HDI was defined as the mean HDI during the study period.

### Statistical analysis

Correlations between diabetes prevalence and national HDI were assessed via linear regression analysis. The Student's *t-test* was used to compare categorical variables between two HDI groups. Comparisons between multiple groups were conducted using one-way ANOVA followed by the Tukey-Kramer post hoc test. Statistical analysis was performed using SPSS 17.0 (IBM, Chicago, IL, USA). Data were plotted using GraphPad Prism 5 (GraphPad, San Diego, CA, USA).

## SUPPLEMENTARY MATERIALS TABLE




