# Supplementary material for: Socioeconomic status is associated with global diabetes prevalence
Source: Oncotarget. 2017 May 16;8(27):44434–9. doi: 10.18632/oncotarget.17902 (PMC5546491; doi:10.18632/oncotarget.17902)
Supplement: Supplementary file 1 [file oncotarget-08-44434-s001.pdf]

Socioeconomic status is associated with global diabetes prevalence

Supplementary Materials

Supplementary Table 1: characteristics of the included studies. See Supplementary_Table_1
